# Comprehensive dialectical behavior therapy for adolescents in a juvenile correctional treatment center: a pilot evaluation

**DOI:** 10.3389/frcha.2023.1207575

**Published:** 2023-11-03

**Authors:** Johanna B. Folk, Phillip Yang, Anne Thomas, Jayme Lyon, Jaisal Patel, Clara Yoon, Barbara Robles-Ramamurthy

**Affiliations:** ^1^ Department of Psychiatry and Behavioral Sciences, University of California, San Francisco, San Francisco, CA, United States; ^2^ School of Medicine, University of Texas Health San Antonio, San Antonio, TX, United States; ^3^ Bexar County Juvenile Probation Department, San Antonio, TX, United States; ^4^ Department of Psychiatry and Behavioral Sciences, University of Texas Health San Antonio, San Antonio, TX, United States

**Keywords:** dialectical behavior therapy for adolescents, forensic psychology, adolescent, mental health, juvenile justice

## Abstract

**Background:**

Incarcerated youth commonly present with emotion dysregulation, aggression, and comorbid psychiatric disorders, yet often do not receive necessary mental health treatment while confined. It is therefore crucial to expand the evidence base regarding empirically supported mental health interventions which are feasible to implement in secure settings to address incarcerated youth's mental health needs. Through a community-academic partnership, the current pilot study evaluated a comprehensive dialectical behavior therapy program implemented in a juvenile correctional treatment center.

**Methods:**

Youth participants (*N* = 113) were on average 15.37 years old (SD = 1.10, range = 13–17), 68.1% boys, and identified as 69.0% Latinx, 22.1% Black, 8.0% White, and 0.9% Native American. Youth received comprehensive dialectical behavior therapy for adolescents (DBT-A), including individual therapy, skills training groups, family therapy, multi-family skills training groups, and skills coaching in the milieu by direct care staff who participated in extensive training and ongoing consultation team meetings. As part of a facility-designed program evaluation, youth completed a battery of empirically validated assessments of mental health and emotion regulation prior to and following completion of the program.

**Results:**

Results show that comprehensive DBT-A is feasible to implement in a juvenile correctional treatment center and overall, youth improved from pre- to post-treatment in mental health symptoms and emotion regulation, with small to medium effect sizes.

**Conclusion:**

These findings build upon a growing literature showing dialectical behavior therapy is a promising intervention for treating emotion dysregulation and mental health conditions and can be successfully implemented in juvenile forensic settings.

## 1. Introduction

On any given day, approximately 43,580 youth are confined in the U.S. due to involvement with the juvenile or criminal legal systems; these 2017 estimates represent an approximately 60% reduction from a high of 108,802 in 2000 (1). Numerous studies have identified high rates of mental health needs among incarcerated youth, with estimates up to 80% having at least one psychiatric disorder (2–8). Epidemiologic data from incarcerated youth indicate half of boys and almost half of girls meet criteria for a substance use disorder, more than 40% of boys and girls for disruptive behavior disorders, and almost 20% of boys and more than 25% of girls for affective disorders (6, 9). Comorbid psychiatric disorders (e.g., affective, anxiety, behavioral, substance use) are also common; 56.5% of girls and 45.9% of boys meet criteria for two or more disorders (10). Incarcerated youth commonly present with extensive trauma histories (11), aggression and emotional dysregulation (12, 13), and are likely to experience re-incarceration as adults, substance abuse, financial difficulty, adverse health outcomes, and early mortality (14–17).

Incarcerated youth face multiple layers of stress and marginalization, requiring thoughtful implementation of evidence-based practices. Racism, poverty and community violence are some commonly experienced social determinants of health that may precipitate mental health needs, limit access to timely and quality mental healthcare and expedite entry into the legal system (18–24). Implementation of evidence-based interventions must strive to reduce the trauma and violence frequently encountered inside facilities, impacting both youth (e.g., peer-to-peer violence, seclusion as punishment) and staff (e.g., assaults on staff) (25, 26). Facility-wide efforts to reduce trauma exposure, address mental health needs, and prevent future legal involvement are desperately needed.

Dialectical behavior therapy (DBT), which was originally developed to treat adults exhibiting suicidal behavior (27), utilizes acceptance, behavioral change, and cognitive intervention strategies collaboratively with clients to achieve behavioral targets, prioritized by order of severity. Priority treatment targets include behaviors harmful to self and/or others, followed by therapy- and quality of life-interfering behaviors, other symptom relief, achieving individual goals and greater self-respect, in service of building a “life worth living.” DBT teaches adaptive core skills of mindfulness, distress tolerance, interpersonal effectiveness, and emotion regulation. Due to its effectiveness in promoting the development of emotional and behavioral management skills, DBT has been adapted to treat conditions associated with emotional dysregulation [e.g., oppositional defiant disorder, eating disorders; (28–30)] and to build positive emotions, resilience, and social-emotional learning (31, 32).

DBT's adaptation for adolescents (DBT-A) incorporates families into treatment and a middle path skills training module, which seeks to synthesize two seemingly opposite positions common to parent-child relationships [e.g., over-pathologizing normal behavior vs. normalizing pathological behavior; (33)]. To attain optimal results, comprehensive DBT-A includes: (1) individual therapy, (2) group skills training to teach skills and enhance capabilities, (3) coaching to ensure generalization of acquired skills, (4) family DBT “as needed” to teach caregivers skills and for structuring the environment in support of treatment, and (5) consultation teams to provide support, enhance skills, and increase motivation among providers (34).

DBT has also been adapted for incarcerated populations (DBT Corrections-Modified; DBT-CM) (35), with modifications to the manual language and content of “real life” scenarios to make them more understandable and relevant (e.g., vocabulary and pictures for the lower educational level common to incarcerated populations). Skills training also includes plans for after release (36). DBT-CM has been implemented in juvenile correctional settings and found to reduce aggressive behavior, suicidal ideation, and recidivism (36–38).

Comprehensive DBT has better outcomes than without all DBT elements (39). It involves training for youth and staff, which is crucial given evidence that implementation of trauma-informed practices addresses correctional violence only if a certain threshold of both youth and staff are trained (40). A recent scoping review revealed eight published studies evaluating the effectiveness of DBT in secure youth forensic settings (41). Existing literature on DBT within these settings is limited and relies on predominantly small sample sizes of youth (i.e., half had 90 or fewer participants) in single facilities and using within-subjects designs without comparison conditions. No studies reviewed evaluated comprehensive DBT; one intended to, however environmental demands prevented them from doing so (42). This review also documented how implementation of DBT in these settings can be challenging due to lack of resources, lack of staff or staff turnover, or uncertainty about best practices for program implementation (43, 44).

The current pilot study was an academic-community partnership examining a comprehensive DBT-A program implemented in a long-term, post-adjudication juvenile correctional treatment center. The aim was to gather preliminary evidence regarding the impact of participating in comprehensive DBT-A on youth mental health and emotion regulation. It was hypothesized youth would exhibit improvements in emotion regulation and mental health. This pilot study also aims to document one facility's approach to implementing comprehensive DBT-A, in the hopes that lessons learned could guide other facilities interested in developing such programs.

## 2. Method

### 2.1. Facility

Participants resided in a county-operated 96-bed juvenile correctional treatment center serving adolescent boys and girls. Youth are court ordered to this secure facility, typically after multiple adjudications and efforts to provide community-based supervision and treatment. All youth in the facility receive comprehensive DBT-A from a multidisciplinary treatment team; youth can also receive psychiatric care and substance use treatment, as needed. The facility was selected because of the: (1) established multidisciplinary treatment structure which facilitated effective coordination of DBT-A; (2) average length of stay for youth in the facility, which allowed the opportunity to complete all skills training modules (approximately 6 months); and (3) population of English-speaking youth who had been difficult to engage in lesser intensive settings and with other treatment modalities but had not yet been convicted of an aggravated crime which typically would lead to the commitment to the state juvenile system.

### 2.2. Program

In 2017, the county obtained grant support to acquire intensive training and consultation in the implementation of comprehensive DBT-A. DBT-A was selected due to its developmental appropriateness (e.g., skills training to help adolescents resolve age specific challenges, incorporates family); the curriculum was adapted to include elements of DBT-CM and DBT for substance use disorders to meet the specific needs of incarcerated youth.^1^

#### 2.2.1. Staff training

All participating administrators, medical, educational, mental health and direct care staff participated in a yearlong intensive training schedule including: (1) introduction of the DBT-A model to executive leadership during one 8-hour session; (2) four 8-hour sessions with clinicians on utilizing DBT-A within individual, group, and family sessions; (3) five 8-hour sessions with clinicians, correctional supervisors, and direct care staff on implementing skills training modules within the milieu; (4) five different consultation teams discussing implementation issues with the expert trainer for 2 h per month over 6 months; and (5) another 8-hour session with executive management to review progress and formulate future plans. Ongoing consultation with the DBT trainer and annual booster training for the entire facility has continued since. Regarding turnover, the introductory DBT-A training is offered with each new officer orientation. Two DBT coaches (mid-level correctional staff) also provide shoulder-to-shoulder training and coaching as staff utilize DBT-A in their daily interactions with teens.

#### 2.2.2. DBT-A components

Participating youth received the following services from trained master's level licensed clinicians: (1) weekly individual therapy; (2) weekly skills training groups; (3) bi-weekly family therapy; and (4) multi-family skills training groups. Youth also had access to skills coaching in the milieu by direct care staff, designated DBT coaches, and clinicians. Diary Cards were completed weekly and reviewed in individual therapy. Behavior Chain Analyses were completed regularly for effective and ineffective behaviors. Families were invited to attend biweekly family therapy with their teen, weekly visitation hours, and multifamily DBT focused sessions. Parental attendance was documented for family therapy and multifamily sessions and included in reports to probation and the court with annual participation rates ranging from 80%-92%.

Once youth completed all 18 skills groups and the accompanying homework, they took a DBT Competency Exam and participated in a coaching role play to determine whether they fundamentally grasped DBT-A concepts; in the history of the program, except for two youth, all have passed this exam on their first attempt. Youth then participated in a three-week Crime Review group (adapted from DBT-CM) led by a DBT-A clinician, completing a behavior chain analysis on their referring offense and learning skills that could have been used to interrupt the chain of events. Youth also participated in a Graduation Review presentation in which they acknowledged their accomplishments in placement, shared their therapeutic gains, and discussed their future plans.

Multidisciplinary treatment teams met weekly to address youth's treatment needs, progress, and award incentives. These teams ensured youth made progress toward treatment targets and directed adaptations as needed to meet any individual needs. Treatment targets on diary cards were evaluated to ensure they were realistic and attainable, and specialized interventions were developed for additional support in achieving these targets (e.g., individual coaching in the milieu to understand instructions and manage transitions and feelings in the moment). Clinicians and direct care staff utilized DBT-A strategies such as validation, “foot in the door” and “pros and cons” to encourage willingness to engage in treatment. With full recognition of the external pressures experienced by youth as the result of incarceration and court-mandated requirements for release, it was especially important to avoid coercive demands for compliance in favor of an emphasis on acceptance and validation.

Consultation Teams provide emotional support and validation for direct care staff and mental health providers, help ensure treatment fidelity, improve provider capabilities and reduce burn-out (45, 46). Mental health providers had regular consultation team meetings and the Staff Education and Support Meeting functioned as the Consultation Team for direct care staff; correctional supervisory staff regularly attended and direct care staff rotated attendance due to shift schedules (47). Clinicians facilitated these meetings to validate experiences working with youth and promote learning and practicing DBT-A skills which they, in turn, can offer as they coach youth within the milieu.

The facility's behavioral intervention plan was improved to align more generally with DBT-A principles and emphasize supportive and trauma informed approaches. The reward system focused on encouraging skillful behavior through increasing privileges and autonomy and discouraging unskillful behavior through consequences designed to expand learning opportunities. Youth exhibiting harmful or severely program disruptive behavior requiring separation from the general population were expected to follow the Egregious Behavior Protocol; they completed a behavior chain analysis of the incident and reviewed it with staff to identify unique vulnerabilities and triggers leading to the incident and to determine specific skills or behavior necessary to prevent future incidents. Youth remained on protocol until completion of the repair consisting of correction/overcorrection. Youth corrected damage caused by their behavior (e.g., fixing or paying for property they destroyed, working to repair damage to relationships) and overcorrected by identifying, learning, and practicing a new behavior or skill designed to break the old behavioral pattern.

### 2.3. Participants

Youth included in the current analysis (*N* = 212) participated in the comprehensive DBT-A program from its 2017 inception through October 2021. Youth who completed pre- and post-tests (*n* = 113) were the primary analytic sample. Youth who were missing post-test information were not included in primary analyses (*n* = 99); these youth did not significantly differ from those included on any demographic factors or primary variables of interest at pre-test. Type of discharge differed between those included (92.0% successful, 3.5% unsuccessful, 0.9% alternate placement, 3.5% probation expired) and excluded (65.7% successful, 26.3% unsuccessful, 1.0% alternate placement, 4.0% probation expired, 3% unknown). Excluded youth were significantly more likely to have an unsuccessful discharge and included youth were significantly more likely to have a successful discharge (*X^2^* = 23.86, *p* < .001); alternate placement and probation expired were not included in the comparison given the small cell sizes.

### 2.4. Measures

Eight master's level clinicians (full-time facility employees delivering DBT-A) administered the following self-report measures within 30 days of admission (pre-test) and 30 days before discharge (post-test). Clinicians also recorded youth demographic information.

#### 2.4.1. Difficulties in emotion regulation scale short form (DERS-Sf)

The DERS-SF (48) is an 18-item assessment of an individual's ability to manage feelings. Responses are rated from 1 (almost never) to 5 (almost always) and averaged to create six, three-item subscales: strategies (*α*: pre = .49, post = .72), non-acceptance (*α*: pre = .72, post = .76), impulse (*α*: pre = .88, post = .90), goals (*α*: pre = .86, post = .86), awareness (*α*: pre = .74, post = .82), and clarity (*α*: pre = .66, post = .65); item level data to calculate alphas was only available for a subset of youth (*n* = 80–96 depending on subscale and timepoint) and the remainder had subscale scores only. Higher scores reflect greater emotion dysregulation.

#### 2.4.2. Beck youth inventories, 2nd edition (BYI-2)

The BYI-2 (49) is a 100-item assessment of emotional and social impairments. Responses are rated from 0 (never) to 3 (always) and summed to create raw totals for five, 20-item subscales: self-concept, anxiety, depression, anger, and disruptive behavior. Raw scores are converted to t-scores using age- and gender-based norms. T-scores are interpreted as: 55 or less = average, 55–59 = mildly elevated, 60–69 = moderately elevated, and 70+=extremely elevated; for self-concept, t-scores in the elevated ranges reflect more positive self-concept compared to peers. BYI-2 data was only available for a subset of the sample (approximately 40%) primarily due to measure supply shortages. Item-level data used to calculate alphas was available for only a subset of those youth (*n* = 29–34 depending on subscale and timepoint). Alphas at pre- and post-test, respectively are: self-concept (.88,.88), anxiety (.92,.88), depression (.92,.93), anger (.94,.92), and disruptive behavior (.88,.88).

#### 2.4.3. Adolescent anger rating scale (AARS)

The AARS (50) is a 41-item assessment of anger responses. Responses are rated from 1 (hardly ever) to 4 (very often) and summed to created three subscales: Instrumental Anger (20 items), Reactive Anger (8 items), and Anger Control (13 items); the raw total for the subscale anger control is subtracted from the sum of the instrumental and reactive anger subscales to obtain a total anger subscale. Although the assessment has grade- and gender-based norms, the current study compared the raw total scores because: (1) youth in the facility are on average 2 grades behind their same-age peers, often due to frequent moves and truancy (based on facility data), and (2) the grade level of most youth was not recorded at the time the measures were administered. AARS data was only available for a subset of the sample (approximately 61%) also because of measure supply shortages. Alphas at pre- and post-test, respectively are: instrumental anger (.84, .85), reactive anger (.85, .82), and anger control (.75, .82).

#### 2.4.4. Trauma symptom checklist for children (TSCC)

The TSCC (51) is a 54-item measure of youth posttraumatic symptomatology. Items are rated from 0 (never) to 3 (almost all the time), summed to create raw scores, and converted to t-scores using age- and gender-based norms. Clinical scales include: Anxiety (9 items; *α*: pre = .80, post = .70), Depression (9 items; *α*: pre = .84, post = .76), Anger (9 items; *α*: pre = .89, post = .82), Posttraumatic Stress (10 items; *α*: pre = .89, post = .82), Dissociation (10 items; *α*: pre = .85, post = .81), and Sexual Concerns (10 items; *α*: pre = .73, post = .74). Twenty-two items are included in multiple subscales. The TSCC also includes validity scales assessing under- and hyper-reporting.

#### 2.4.5. Juvenile victimization questionnaire, 2nd revision, reduced item (JVQ-R2)

The JVQ-R2 (52) is a 12-item shortened version of the original Juvenile Victimization Questionnaire (53). Youth respond yes or no to each item asking if they have experienced the following types of victimization: emotional maltreatment (2 items), witnessing (3 items), physical maltreatment (4 items), property assault (1 item), and sexual victimization (2 items). The cutoff for polyvictimization is 5 or more endorsed items.

### 2.5. Plan of analysis

All analyses were conducted using SPSS v.28 statistical software. Preliminary analyses included descriptive statistics of all study variables. Dependent samples t-tests were used to assess pre/post changes in emotion regulation and mental health symptoms. Cohen's d was calculated as a measure of effect size and is interpreted following Cohen's (1988) established guidelines (0.20 = small, 0.50 = medium, 0.80 = large) (54).

We also examined whether pre-test demographic and mental health characteristics were related to type of discharge from the program (successful vs. unsuccessful). These analyses incorporated the full sample for whom type of discharge was known (*n* = 198). Chi-square tests were used for categorical variables (e.g., gender, race and ethnicity) and t-tests for continuous variables (e.g., mental health variables, age, length of stay).

## 3. Results

### 3.1. Sample characteristics

Youth (*n* = 113) were on average 15.37 years old (*SD* = 1.10, range = 13–17), 68.1% boys, and identified as 69.0% Latinx, 22.1% Black, 8.0% White, and 0.9% Native American. At pre-test, 95% of youth reported lifetime trauma exposure. Additional characteristics are presented in Table 1.

**Table 1:** mental health and trauma exposure characteristics of juvenile correctional center youth participants (2017-2021).

Characteristic	Descriptive statistic
Trauma history	
Trauma exposure, *M(SD), range*	6.39 (2.71), 0–12
Polyvictimization (% who endorsed 5 + items)	77%
Beck youth inventories clinical elevations, % (*n*)	
Anxiety	26% (*n* = 16 of 62 responses)
Depression	31% (*n* = 19 of 61 responses)
Anger	34% (*n* = 21 of 61 responses)
Disruptive behavior	59% (*n* = 36 of 61 responses)
Self-concept	34% (*n* = 21 of 62 responses)
Trauma symptom checklist for children clinical elevations, % (*n*)	
Anxiety	9% (*n* = 10 of 107 responses)
Depression	9% (*n* = 9 of 98 responses)
Anger	8% (*n* = 9 of 107 responses)
Posttraumatic stress	14% (*n* = 15 of 107 responses)
Dissociation	18% (*n* = 19 of 107 responses)

### 3.2. Changes in emotion regulation and mental health symptoms

Results are displayed in Table 2. Improvements in emotion regulation were observed on the DERS-SF, in all subscales except non-acceptance, representing small to medium effects (*d*'s = 0.20–0.37). Improvement in anger control on the AARS was also observed, with medium effect sizes for total, instrumental, and reactive anger (*d* = 0.50–0.68) and a small effect (*d* = .21) for anger control.

**Table 2:** Changes in emotion regulation and mental health symptoms following participation in a comprehensive DBT program.

Assessment	*n*	Pre-test	Post-test	Difference		*t*	d
		*M* (*SD*)	*M* (*SD*)	*M* (*SD*)	95% CI		
Difficulties in emotion regulation scale							
Strategies	110	2.24 (0.98)	1.86 (0.86)	0.39 (1.04)	0.19, 0.58	3.91***	0.37
Non-acceptance	110	1.80 (0.91)	1.67 (0.89)	0.13 (1.11)	−0.08, 0.34	1.26	0.12
Impulse	110	2.78 (1.18)	2.32 (1.16)	0.46 (1.42)	0.19, 0.73	3.40***	0.32
Goals	110	3.18 (1.19)	2.85 (1.09)	0.33 (1.33)	0.07, 0.58	2.58*	0.25
Awareness	110	2.94 (1.09)	2.56 (1.15)	0.38 (1.14)	0.16, 0.59	3.48***	0.33
Clarity	110	2.10 (0.91)	1.92 (0.85)	0.18 (0.91)	0.01, 0.35	2.05*	0.20
Adolescent anger rating scale							
Total	66	90.83 (16.37)	80.95 (13.94)	9.88 (17.07)	5.68, 14.08	4.70***	0.58
Instrumental anger	67	33.46 (8.61)	28.72 (6.95)	4.75 (9.55)	2.42, 7.08	4.07***	0.50
Reactive anger	67	19.84 (5.93)	16.25 (5.11)	3.58 (5.28)	2.30, 4.87	5.56***	0.68
Anger control	67	27.01 (7.47)	28.84 (7.31)	−1.82 (8.52)	−3.90, 0.26	−1.75*	0.21
Trauma symptom checklist for children							
Anxiety	104	49.96 (10.59)	46.85 (7.78)	3.12 (10.76)	1.02, 5.21	2.95**	0.29
Depression	104	49.13 (9.28)	46.05 (7.56)	3.08 (9.09)	1.31, 4.84	3.45***	0.34
Anger	104	49.82 (9.76)	45.32 (7.21)	4.50 (9.94)	2.57, 6.43	4.62***	0.45
Posttraumatic stress	103	52.31 (11.26)	49.53 (9.88)	2.78 (10.33)	0.76, 4.80	2.73**	0.27
Dissociation	103	52.72 (12.20)	49.73 (10.08)	4.28 (11.46)	2.04, 6.52	3.79***	0.37
Sexual concerns	102	50.56 (15.82)	48.72 (11.79)	1.84 (13.98)	−0.90, 4.59	0.18	0.07
Beck youth inventories							
self-concept	44	44.18 (12.13)	49.95 (9.32)	−5.77 (12.95)	−9.71, −1.84	−2.96**	0.45
Anxiety	44	55.68 (10.90)	51.36 (8.95)	4.32 (10.31)	1.18, 7.45	2.78**	0.42
Depression	43	53.30 (9.81)	48.91 (7.93)	4.40 (9.53)	1.46, 7.33	3.02**	0.46
Anger	43	56.40 (11.95)	51.49 (9.68)	4.91 (13.59)	0.73, 9.09	2.37*	0.36
Disruptive behavior	43	63.93 (12.10)	56.98 (11.44)	6.95 (13.62)	2.76, 11.15	3.35***	0.51

* *p* < .05.

** *p* < .01.

*** *p* < .001.

Improvements in mental health were observed on all TSCC domains except sexual concerns, representing small to medium effect sizes (*d*'s = 0.20–0.45). Most youth (72.6%, *n* = 77) produced valid TSCC responses, with no significant elevations on the under or overreporting subscales at pre- or post-test. At pre-test, 15.0% of respondents were elevated on the underreporting subscale and 3.7% on the overreporting subscale. At post-test, 17.3% of respondents were elevated on underreporting and 0.9% on overreporting. Analyses of TSCC data were conducted excluding respondents with elevations on validity scales; findings were consistent with analyses of the full sample, with the exception sexual concerns. Anxiety [*t*(76) = 1.90, *p* = .030, *d* = 0.22], depression [*t*(76) = 2.84], *p* = .003, *d* = 0.32), anger, *t*(76) = 4.05, *p* < .001, *d* = 0.46), posttraumatic stress [*t*(76) = 2.35, *p* = .011, *d* = 0.27], and dissociation [*t*(76) = 3.62], *p* < .001, *d* = 0.41) all improved; no changes were observed in sexual concerns [*t*(75) = 0.18, *p* = .276, *d* = 0.07].

Improvements in mental health on all domains of the BYI were also observed, reflecting small to medium effects (*d*’s = .36-0.51).

### 3.3. Characteristics related to type of discharge

Boys were significantly more likely to be unsuccessfully discharged and girls more likely to be successfully discharged than expected (*X^2^* = 4.05, *p* = .044). There were no significant differences in type of discharge based on age, race, ethnicity, length of stay in the facility, or any mental health characteristic at pre-test.

## 4. Discussion

The current pilot study evaluated a comprehensive DBT-A program in a juvenile correctional treatment center. Consistent with prior studies of DBT in juvenile incarceration settings, improvements in mental health symptoms and emotion regulation were observed from pre- to post-treatment (36, 55, 56). This is promising given the documented high rates of mental health symptoms and disorders among incarcerated youth, as well as their elevated risk for suicide (57). Furthermore, evidence indicates improving emotion regulation during incarceration reduces the likelihood of youth recidivism (58). These findings build upon a growing literature showing DBT is a promising intervention for treating emotion dysregulation and can be successfully implemented in juvenile forensic settings (28–30).

Implementation required significant resources and commitment from the county and facility. Prior studies show comprehensive DBT can be difficult to implement in juvenile correctional settings due to lack of resources, insufficient staffing, and uncertainty about how best to implement the program (43, 44). Implementation was costly due to the scarcity of expert training available, need for intensive initial training followed by ongoing training and consultation to minimize learning loss and consultation to ensure fidelity, and the importance of acquiring essential therapeutic resources (e.g., trauma-informed care intervention tools, incentives); for many facilities this approach is cost prohibitive.

The institution's multidisciplinary organizational structure was crucial to facilitating implementation, however this may not be standard in other facilities. Comprehensive DBT can be challenging to implement due to limited communication and coordination of interventions by mental health and correctional staff (59). Put simply, mental health staff often incorporate acceptance-based interventions (e.g., increasing self-awareness, validating emotions) whereas correctional staff interventions are generally change-based (e.g., environmental structure using a plan of contingencies to modify behavior). Comprehensive DBT is unique in seeking to enhance mutual understanding between these two seemingly opposite approaches, as illustrated by one of its fundamental assumptions: People may not have caused all their problems (acceptance), yet they must solve them anyway (change).

Successful comprehensive DBT-A implementation required a commitment to truly collaborative work to create fully functioning multidisciplinary teams sharing the goal of accepting the youths’ experiences, perspectives, feelings and thoughts as valid while simultaneously helping them navigate systems of accountability. The legal system has not demonstrated effectiveness in reducing recidivism or improving youth wellbeing, so clinicians and correctional staff can use DBT to balance acceptance of limitations and work toward necessary change of the systems they uphold. Building effective communication to overcome distrust and improve collaboration between treatment and corrections professionals is addressed specifically in consultation team meetings. Effective DBT multidisciplinary teams work to find solutions so youth obtain the intended benefit of the treatment tool while at the same time, minimize potential risks of harm to self and others within the facility.

### 4.1. Strengths, limitations, and future directions

This study adds to the literature as it is the first evaluation, to our knowledge, of comprehensive DBT-A in a secure youth forensic setting. Strengths include use of empirically validated assessments, inclusion of girls and boys and an ethnoracially diverse sample, and a larger sample than most prior studies evaluating DBT in secure youth incarceration settings (41). This pilot study also reflects real-world challenges to both the empirical analysis of treatment effects as well as the implementation of a comprehensive DBT-A treatment model in a juvenile correctional setting. First, the study relied on archival self-report data collected as part of a program evaluation involving a pre-post design and no comparison condition. This may limit confidence in the findings as change in outcomes could be due to maturation or timing effects or to other interventions received (e.g., psychiatric or substance use services). Federal protections for research with incarcerated youth necessarily limit the use of control groups, however future research should consider alternate designs (e.g., interrupted time series) to examine effectiveness (60). Although rigorous and valuable, randomized control trials are not always possible and alternate designs can provide useful evidence regarding the effectiveness of interventions (61). Future research should also incorporate behavioral (e.g., frequency of self-harm behavior) and collateral data (e.g., incident reports, frequency of restraint use) to understand the full impact of participation in DBT-A in a correctional setting.

Second, it is possible the sample was biased toward documenting improvements, as youth with available post-treatment data were more likely to be successfully discharged than those with only pre-treatment data. Discharge decisions are complex and treatment length therefore is individualized. Future studies should maximize post-treatment data collection from youth with unsuccessful discharges, including by providing opportunities to complete assessments post-release or in new placement settings.

Third, the current sample was from a single facility and impacted by the available resources. Running out of paid assessment measures and human-error (e.g., missing the backside of double-sided forms, youth refusal to complete full assessment batteries) resulted in missing data. Data gathering, management and analysis could be strengthened by establishing early partnerships with academic centers. Fidelity was supported through consultation team meetings and DBT coaches assisting staff, but not monitored systematically, a consideration for future studies.

Fourth, although our sample was racially diverse, analyses were not sufficiently powered to examine differential effectiveness of the intervention on youth from different ethnoracial backgrounds. Detailed data on youth ethnicity, beyond whether they identified as Latinx, was also unavailable. Future research should explore whether youth from certain ethnoracial groups and other identities (e.g., gender) benefit differentially from DBT-A.

Fifth, additional research is needed to examine the long-term effects of comprehensive DBT-A on youth and society more broadly. Our study was limited to examining changes in mental health and emotion regulation during incarceration; future research should examine impacts on post-incarceration outcomes such as recidivism, family cohesion and linkage to ongoing mental health treatment, as well as on staff burnout, turnover, and secondary traumatic stress. Finally, future research should consider the social and monetary cost of implementing comprehensive DBT-A in correctional settings as opposed to in the community.

## 5. Conclusion

Findings from the current study build upon a growing literature showing DBT-A is a promising intervention well-suited for treating emotion dysregulation and mental health conditions. With careful attention to the context of youth incarceration (e.g., punitive and mandated nature of incarceration, need for collaboration with legal system direct care staff), comprehensive DBT-A can be successfully implemented in secure juvenile forensic settings.

## Data Availability

Due to the sensitive nature of the data collected from incarcerated youth, data is not publicly available. Requests to access the datasets should be directed to Johanna Folk, johanna.folk@ucsf.edu.

## Footnotes

^1^Additional details regarding the implementation plan, training materials, and adapted intervention materials are available upon request to Anne Thomas (athomas1234@att.net).
